# Supplementary material for: 3-Bromopyruvate Suppresses the Malignant Phenotype of Vemurafenib-Resistant Melanoma Cells
Source: Int J Mol Sci. 2022 Dec 9;23(24):15650. doi: 10.3390/ijms232415650 (PMC9779063; doi:10.3390/ijms232415650)
Supplement: Supplementary file 1 [file ijms-23-15650-s001.zip › ijms-1804473-supplementary.pdf]

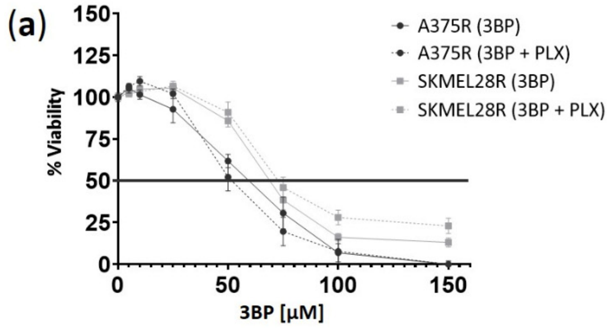

(b)

Cell line	Treatment	48 h IC_{50} (μM)
A375R	3BP	53.12 ± 5.32
	3BP + PLX	52.23 ± 5.02
SKMEL28R	3BP	70.11 ± 3.30
	3BP + PLX	57.18 ± 1.93

Figure S1. Cell viability analysis using a sulforhodamine B (SRB) assay in PLX-resistant melanoma cells treated with 3-bromopyruvate (3BP) for 48 hours. (**a**) The dose-response curve represents the viability of vemurafenib (PLX)-resistant melanoma cells treated with different concentrations of 3BP (0-150 μM), either alone or combined with PLX (4.5 μM for A375R cells and 6.0 μM for SKMEL28R cells). Data were normalized to the vehicle control (100%). (**b**) IC_{50} values of PLX-resistant melanoma cells treated with 3BP for 48 hours. Data represent the mean ± SEM of at least three independent experiments performed in triplicate.

(a)

IC_{50} Values (24 h)				
**Cell line**	**PLX**	**3BP**	**3BP + 4.5 μM PLX**	**3BP + 6.0 μM PLX**
**A375R**	8.14 ± 2.00	61.11 ± 6.52	50.30 ± 1.51	-
**SKMEL28R**	ND	83.11 ± 2.47	-	94.09 ± 4.52
**A375**	3.65 ± 0.52	21.72 ± 1.16	29.80 ± 3.60	-
**SKMEL28**	5.54 ± 0.51	57.18 ± 1.93	-	95.60 ± 9.20

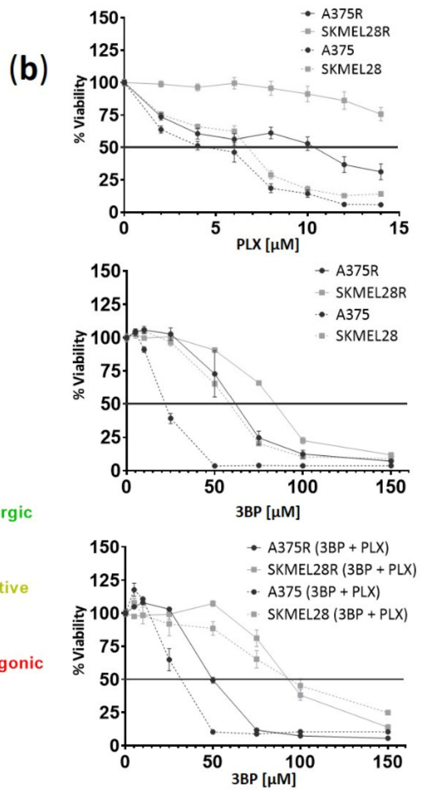

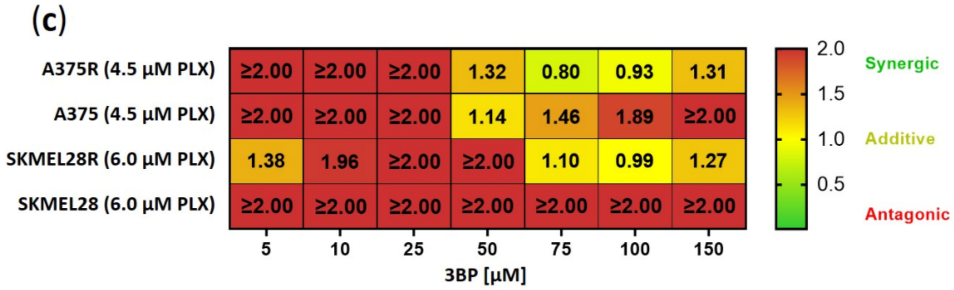

Figure S2. Combination assay and cell viability analysis using a sulforhodamine B (SRB) assay in naïve and vemurafenib (PLX)-resistant melanoma cells treated with PLX, 3-bromopyruvate (3BP), and 3BP+PLX for 24 hours. (**a**) IC_{50} values of naïve and PLX-resistant melanoma cells treated with 0-14 μM PLX or 0-150 μM 3BP alone or in combination with PLX (4.5 μM for A375R cells and 6.0 μM for SKMEL28R cells) for 24 hours. ND, not determined. (**b**) The dose-response curve represents the viability of naïve and PLX-resistant melanoma cells treated with different concentrations of 3BP, PLX, and 3BP+PLX. Data were normalized to the vehicle control (100%). Data represent the mean ± SEM of at least three independent experiments performed in triplicate. (**c**) Combination index (CI) heatmap of melanoma cell lines treated with 0-150 μM 3BP and PLX (4.5 μM for A375 and 6.0 μM for SKMEL28) for 24 hours. CI > 1 antagonism or competition; CI = 1 additive; CI < 1 synergism.

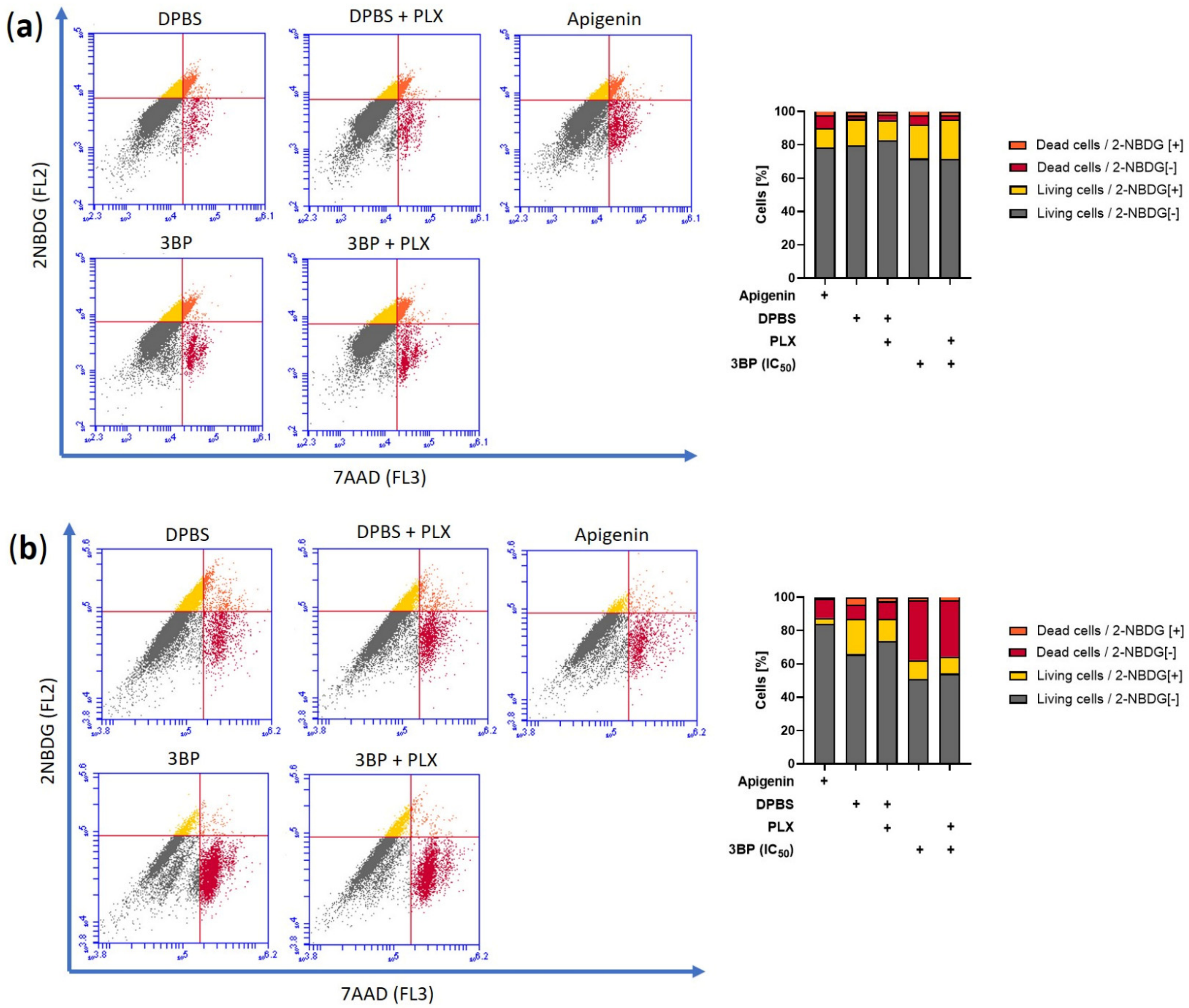

Figure S3. Flow cytometry analysis of A375R and SKMEL28R melanoma cells treated with 3-bromopyruvate (3BP) after 7-aminoactinomycin D (7AAD)/ 2-(N-(7-nitrobenz-2-oxa-1,3-diazol-4-yl)amino)-2-deoxyglucose (2NBDG) staining. (**a**) Representative flow cytometry plots of the A375R cell line treated with apigenin, Dulbecco's phosphate-buffered saline (DPBS), or 3BP, either alone or combined with 4.5 μM vemurafenib (PLX), for 4 hours and a chart summarizing the percentage distribution of different categories of cell death. (**b**) Representative flow cytometry plots of the SKMEL28R cell line treated with 3BP, either alone or combined with 6.0 μM PLX, for 4 hours and a chart summarizing the percentage distribution of different categories of cell death. Flow cytometry profiles represent 7AAD staining on the *x*-axis and 2NDBG on the *y*-axis. The positive signs in the charts indicate the presence of a defined treatment. Data represent the means of at least three independent experiments performed in triplicate.

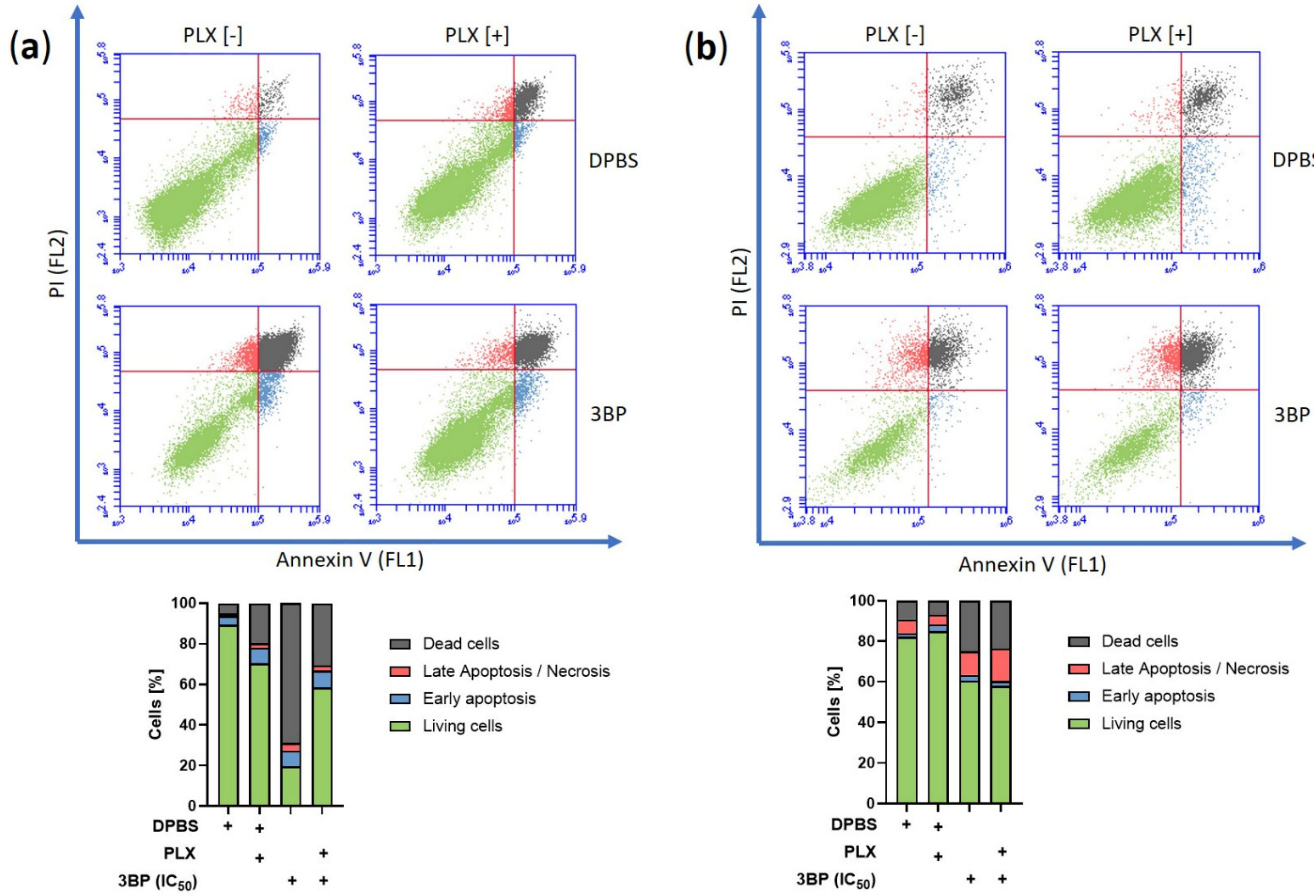

Figure S4. Flow cytometry analysis of A375R and SKMEL28R melanoma cells treated with 3-bromopyruvate (3BP) after annexin V/propidium iodide (PI) staining. (**a**) Representative flow cytometry plots of the A375R cell line treated with DPBS or 3BP, either alone or combined with 4.5 μM PLX, for 24 hours and a chart summarizing the percentage distribution of different categories of cell death. (**b**) Representative flow cytometry plots of the SKMEL28R cell line treated with 3BP, either alone or combined with 6.0 μM PLX, for 24 hours and a chart summarizing the percentage distribution of different categories of cell death. Flow cytometry profiles represent annexin V staining on the *x*-axis and PI staining on the *y*-axis. The positive signs in the charts indicate the presence of a defined treatment. Data represent the means of at least three independent experiments performed in triplicate.

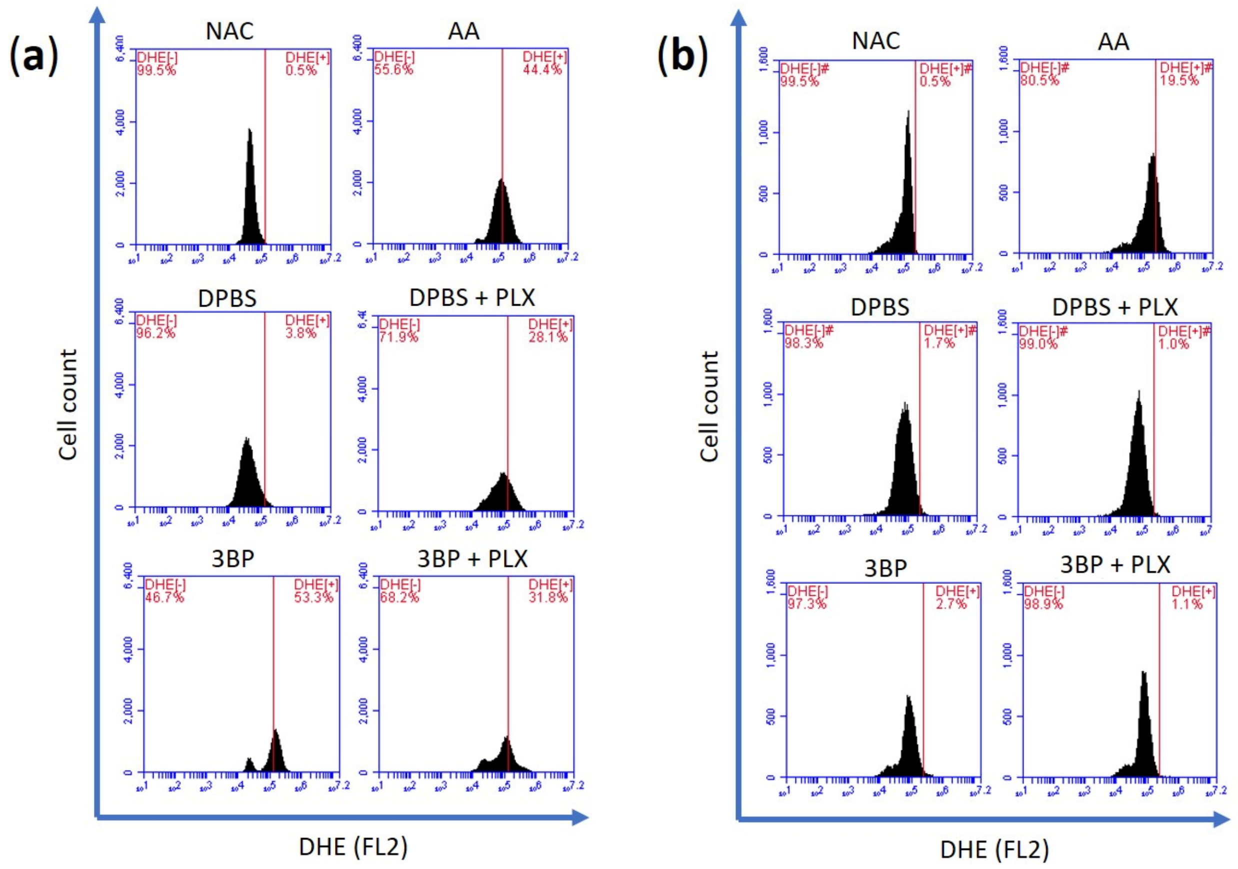

Figure S5. Flow cytometry analysis of A375R and SKMEL28R melanoma cells treated with 3BP after dihydroethidium (DHE) staining. (**a**) Representative flow cytometry plots of the A375R cell line treated with n-acetyl cysteine (NAC), antimycin A (AA), Dulbecco's phosphate-buffered saline (DPBS) or 3-bromopyruvate (3BP), either alone or combined with 4.5 μM vemurafenib (PLX), for 24 hours. (**b**) Representative flow cytometry plots of SKMEL28R cells treated with n-acetyl cysteine (NAC), antimycin A (AA), DPBS or 3BP, either alone or combined with 6.0 μM PLX, for 24 hours. Flow cytometry profiles represent DHE staining on the *x*-axis and cell count on the *y*-axis.

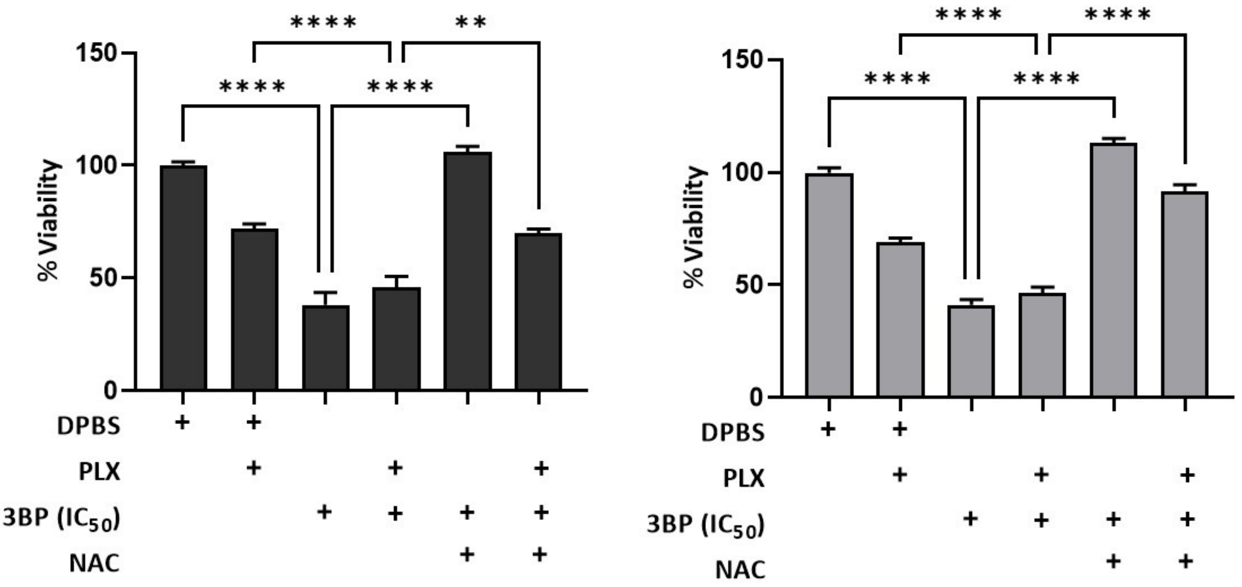

Figure S6. Influence of NAC (N-acetylcysteine) on the viability of vemurafenib (PLX)-resistant melanoma cells. Cell viability analysis using a sulforhodamine B (SRB) assay in PLX-resistant melanoma cells treated with 3-bromopyruvate (3BP) IC_{50}, either alone or combined with PLX (4.5 μM for A375R and 6.0 μM for SKMEL28R), for 24 hours after exposure to 1 mM NAC. Dark gray represents A375R cells, and light gray represents SKMEL28R melanoma cells. Data were normalized to the vehicle control (100%). The positive signs in the bar charts indicate the presence of a defined treatment. Data represent the mean ± SEM of at least three independent experiments performed in triplicate. ** p≤.01; *** p≤.01; **** p≤.0001.
